# Pharmacotherapies of NAFLD: updated opportunities based on metabolic intervention

**DOI:** 10.1186/s12986-023-00748-x

**Published:** 2023-07-06

**Authors:** Yaodi Shao, Suzhen Chen, Liu Han, Junli Liu

**Affiliations:** Shanghai Key Laboratory of Diabetes Mellitus, Shanghai Diabetes Institute, Department of Endocrinology and Metabolism, Shanghai Sixth People’s Hospital Affiliated to Shanghai Jiao Tong University School of Medicine, Shanghai, 200233 China

**Keywords:** Non-alcoholic fatty liver disease, Metabolism, Pharmacological target

## Abstract

Non-alcoholic fatty liver disease (NAFLD) is a chronic liver disease that is becoming increasingly prevalent, and it ranges from simple steatosis to cirrhosis. However, there is still a lack of pharmacotherapeutic strategies approved by the Food and Drug Administration, which results in a higher risk of death related to carcinoma and cardiovascular complications. Of note, it is well established that the pathogenesis of NAFLD is tightly associated with whole metabolic dysfunction. Thus, targeting interconnected metabolic conditions could present promising benefits to NAFLD, according to a number of clinical studies. Here, we summarize the metabolic characteristics of the development of NAFLD, including glucose metabolism, lipid metabolism and intestinal metabolism, and provide insight into pharmacological targets. In addition, we present updates on the progresses in the development of pharmacotherapeutic strategies based on metabolic intervention globally, which could lead to new opportunities for NAFLD drug development.

**Supplementary Information:**

The online version contains supplementary material available at 10.1186/s12986-023-00748-x.

## Introduction

With well over 25% of the world’s population suffering from non-alcoholic fatty liver disease (NAFLD), it is currently the most prevalent chronic liver disease worldwide [1]. Moreover, it is proposed that the NAFLD population in China will increase by 29.1% to 314.58 million during 2016–2030 [2].

**Fig. 1:**
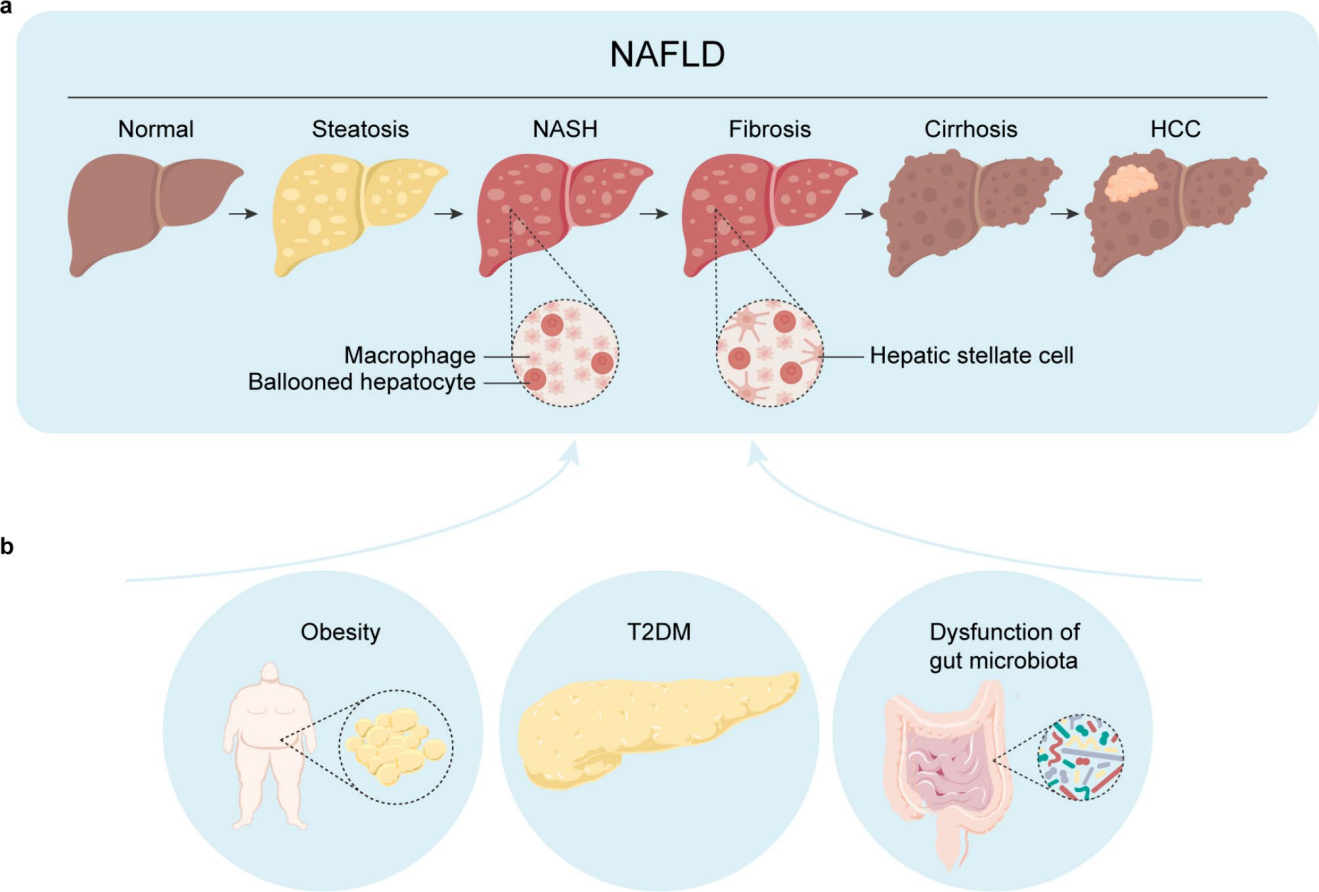
Multiple metabolic dysfunctions contribute to the progression of non-alcoholic fatty liver disease (NAFLD). **a** NAFLD is defined as intrahepatic triglyceride content exceeding 5.5% within hepatocytes and has a sophisticated clinicopathological classification system [8]. Gradually, excessive lipid levels could overwhelm the capacity to deal with inflammation and hepatocyte ballooning due to lipotoxicity, which are characteristic of non-alcoholic steatohepatitis (NASH). Progressively, hepatic stellate cells are actively responsible for inflammation and hepatocyte death. This results in fibrosis through the generation of fibrogenic myofibroblasts [221], and 22% of patients develop cirrhosis [4]. Finally, patients with severe cirrhosis patients progress to hepatocellular carcinoma (HCC). **b** Available evidence indicates that multiple metabolic dysfunctions, such as obesity, type 2 diabetes mellitus (T2DM) and dysfunction of the gut microbiota, are the main risk factors for the progression of NAFLD [3]

The development of NAFLD is progressive with a sophisticated clinicopathological classification system. Individuals with NAFLD mostly present hallmarks of steatosis. In 60% of NAFLD patients, non-alcoholic steatohepatitis develops and is associated with inflammatory infiltration and significant fibrosis [3]. Over time, 22% of NASH-related fibrosis patients progress to cirrhosis, and 2% progress to hepatocellular carcinoma [3][4]. The risk of cardiovascular conditions and malignant carcinoma associated with mortality is increased in individuals with NAFLD (Fig. 1a) [5, 6].

However, patients with NAFLD are typically asymptomatic until the disease progresses to cirrhosis [7]. Initially, symptoms of right upper quadrant pain and fatigue are most commonly noticed. Then, excessive triglyceride accumulation in the liver is detected by imaging examination [8], and increased levels of liver-related enzymes, alanine aminotransferase (ALT) and aspartate aminotransferase (AST), in serum typically reflect hepatocellular damage [9]. The clinical strategy is limited to ameliorating progression through diet modification and exercise; this strategy improves only simple steatosis due to the unsustainability of long-term intervention [10, 11]. In addition, while liver transplants are a reliable treatment for NASH, they are highly expensive, difficult to obtain and carry traumatizing risks. Thus, potential drugs that can replace this treatment in clinical practice are urgently needed [12]. Unfortunately, to date, there is still a lack of clinically approved drugs targeting NAFLD.

The metabolic disorders associated with NAFLD are characterized by dysregulation of lipid metabolism, glucose homeostasis [13] and intestinal-hepatic crosstalk [14], supporting the movement to rename NAFLD as metabolic-associated fatty liver disease (MAFLD) [15]. Moreover, it is helpful to decelerate the progression of NAFLD by improving whole-body metabolic homeostasis to improve associated conditions, such as diabetes and hypertension [16].

In this article, we mainly focus on the metabolic characteristics involved in the development of NAFLD, including glucose metabolism, lipid metabolism and intestinal metabolism, and propose some promising targets for further investigation. Moreover, we assess pharmaceutical targets for NAFLD from the perspective of metabolic intervention and development status at present globally, which might provide new drug development prospects.

## Definition of NAFLD

The stages of NAFLD include non-alcoholic fatty liver (NAFL), NASH, liver fibrosis and liver cirrhosis. NAFLD is first characterized by intrahepatic triglyceride levels exceeding 5.5%, as detected by magnetic resonance spectroscopy or liver biopsy, and the exclusion of secondary causes, such as alcohol abuse, viral infection, other metabolic liver diseases including Wilson’s disease, and drugs, including tamoxifen and amiodarone [8, 17]. Broadly, NAFLD is divided into two pathological forms: NAFL, which shows macrovascular steatosis and mild lobular inflammation, and progressive NASH, which shows ballooning with or without perisinusoidal fibrosis [18]. It is difficult to identify NAFLD in the early stage because the majority of individuals are asymptomatic until they progress to cirrhosis. The most common symptom is right upper quadrant pain, which is then confirmed by ultrasonic evidence or MRI [8]. Consistent with these findings, the serum levels of liver enzymes and albumin are changed with the progression of NAFLD, and these levels reflect whole body dysfunction [19]. Due to the systemic nature of NAFLD, its incidence has been correlated with that of cardiovascular disease, cancer and other conditions, such as chronic kidney disease and obstructive sleep apnea [20]. Patients with severe liver fibrosis are more likely to develop subclinical carotid atherosclerosis, and cardiovascular diseases account for the majority of NAFLD-related mortality [21].

## The metabolic risk of NAFLD

Principally, NAFLD is a systemic disease that can be controlled by whole-body homeostasis, so other diseases, such as polycythemia, hyperuricemia, hypothyroidism, hypopituitarism and polycystic ovary syndrome, could be independent risk factors for its occurrence and development [22–25]. Importantly, the consumption of diets rich in fat and sugar with insufficient exercise may contribute to NAFLD; this may explain the increased prevalence of NAFLD with metabolic impairments [26]. NAFLD is frequently associated with obesity and type 2 diabetes mellitus (T2DM) in China. The proportions of individuals with NAFLD in the obesity and T2DM groups were 60-90% and 28-70%, respectively. Moreover, 51.3% of NAFLD patients had obesity and 22.5% had T2DM [27], which reflects systemic metabolic disorders. Of note, it is increasingly appreciated that the microbiota plays a functional role in regulating metabolic homeostasis, such as that in NAFLD [28], as evidenced by different gut bacteria between obese and lean humans [29] (Fig. 1b).

## Dysregulated metabolism in NAFLD

### Lipid metabolism in NAFLD

In the development of NAFLD, the imbalance between lipid input and output leads to the accumulation of lipids in the liver. Triglycerides (TGs) are the main form of lipids that are stored in the liver and are synthesized by the esterification of free fatty acids (FFAs) [30]. Excessive FFAs impair the liver through lipotoxicity [31–33], mitochondrial dysfunction [34], stimulation of signaling pathways related to metabolism and inflammation [35] and even direct activation of receptors that promote inflammation [36]. Apart from FFAs, intermediates of DNL, such as diacylglycerol, also disrupt metabolic homeostasis [37, 38] through increased reactive oxygen species (ROS) derived from weakening mitochondrial activity [39, 40]. To avoid damage caused by excessive FFAs, the liver will initiate a series of self-protection mechanisms. FFAs can be esterified and transported into serum via very low-density lipoprotein (VLDL). Additionally, FFAs can be oxidized and converted to other substrates. However, in the NASH stage, overwhelmed mitochondria produce ROS, which further aggravates NAFLD [39, 41, 42].

**Fig. 2:**
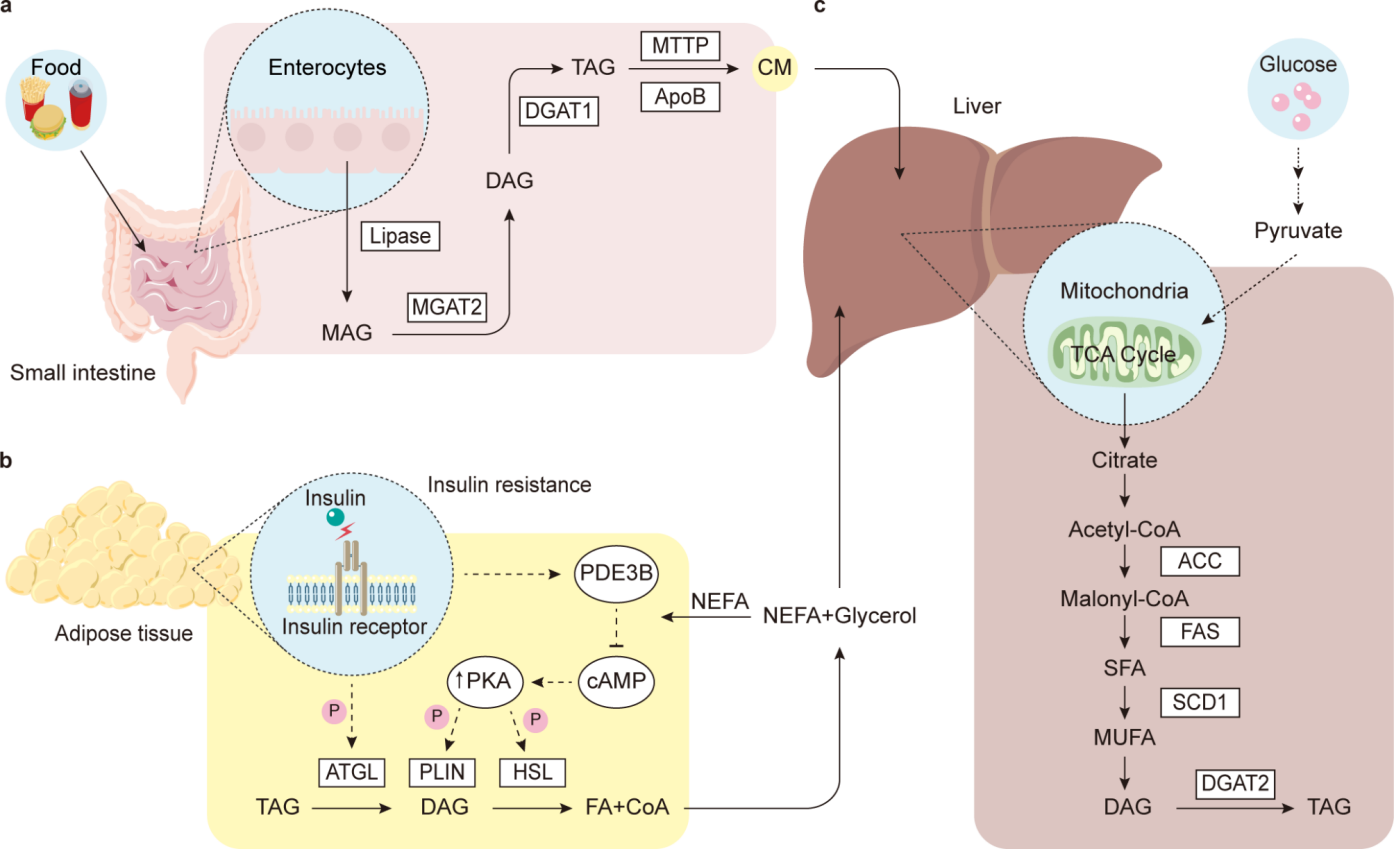
Lipid metabolism in non-alcoholic fatty liver disease (NAFLD). **a**. Under physiological conditions, lipase breaks down triacylglycerol into monoacylglycerol and FFAs, which are then absorbed by intestinal epithelial enterocytes. Then, FFAs and monoacylglycerol are used to resynthesize triacylglycerol by two key enzymatic steps: the first by mannoside acetylglucosaminyltransferase (MGAT) and the second by diglyceride acyltransferase (DGAT). Triacylglycerols are incorporated into chylomicrons (CMs) and secreted into the lymphatic vessels. After catalyzed by lipase, the remnants of CMs absorbed by liver [68, 69]. **b**. Insulin promotes lipid storage by inhibiting lipolysis via adipose triglyceride lipase (ATGL), phosphodiesterase 3B (PDE3B) and protein kinase A (PKA)-controlled hormone-sensitive lipase (HSL) and perilipins (PLINs). However, in regard to insulin resistance conditions (such as obesity or type 2 diabetes mellitus [T2DM]), lower insulin sensitivity stimulates lipolysis, which then leads to more NEFA flux to the liver. **c**. Several key enzymes (such as acetyl-CoA carboxylase [ACC], fatty acid synthase [FAS], stearoyl-CoA desaturase [SCD1] and DGAT2) are involved in de novo lipogenesis in the liver [222]

Considering the role of FFAs in NAFLD, it is vital to understand the three main sources of FFAs. The first is an increase in the spontaneous lipolysis of adipose tissue (59%). The canonical pathway for lipolysis promotes cyclic adenosine monophosphate (cAMP) generation, and then protein kinase A (PKA) is activated to phosphorylate lipases phospho-hormone sensitive lipase (p-HSL) and phospho-perilipin 1 (p-PLIN1). This pathway can be suppressed by insulin [43]. Following their release into circulation, FFAs are taken up by the liver [44]. A number of studies have demonstrated that the lipolysis of adipose tissue in NAFLD, regardless of the existence of diabetes, is increased [45–47]. In obese individuals, due to factors such as adipocyte hypertrophy and insulin resistance, increased lipolysis produces more FFAs, and these FFAs are then transported to the liver (Fig. 2b) [48].

The second source of FFAs is de novo lipogenesis (DNL) (26%). DNL starts with acetyl-CoA subunits, which are mainly derived from glucose [49], and further condensation occurs with the glycerol backbone of these products [50]. There are two major proteins, sterol response element binding protein (SREBP1c) and carbohydrate response element binding protein (ChREBP), that are involved in the transcriptional regulation of DNL [51, 52]. Then, several genes, including fatty acid synthase (FAS), acetyl-CoA carboxylase (ACC) and stearoyl-CoA desaturase 1 (SCD1), are upregulated. Malonyl-CoA is produced from an acetyl-CoA precursor under the controlled catalytic activity of ACC at the beginning of this process [53]. Acyl carrier protein (ACP), which belongs to the FAS domain, transports malonyl-CoA to the prosthetic phosphopantetheine group of the acyl carrier protein [54–56]. Through the prosthetic phosphopantetheine arm of ACP, the elongating FA chain can be shuttled to the different catalytic centers in the active site cleft of FAS by its rotation [57–59]. The malonyl moiety bound to ACP is the additive monomer for elongating the substrate acyl chain, resulting in an elongated 16- or 18-carbon FFA chain [60, 61]. In the initial step of triacylglycerol (TG) synthesis, FFAs are incorporated into glycerol-3-phosphate via primary acylation, resulting in lysophosphatidic acid (LPA) via glycerol-phosphate acyl transferase (GPAT) [50]. In the following step, after desaturated, acylglycerol-phosphate acyl transferase catalyzes LPA to produce phosphatidic acid (PA), which is then dephosphorylated by phosphatidic acid phosphorylase (PAP) to produce diacylglycerol (DG) [62]. Through the catalytic activity of diacylglycerol acyltransferase (DGAT), DG is acylated to TG [63]. DNL not only increases the synthesis of FFAs but also inhibits β-oxidation by its intermediate product malonyl coenzyme (Fig. 2c) [64].

The third source is excessive dietary fatty acids (15%). Hepatocytes take up chylomicron (CM) particle remnants, which contain FFAs [65], and increased absorption of CM remnants leads to the excessive accumulation of lipids in the liver [66, 67]. Mechanically, triacylglycerol is broken down into FFAs and monoacylglycerol by pancreatic lipase. Enterocytes resynthesize triacylglycerol through two sequential acylation steps: first by monoacylglycerol acyltransferase 2 (MGAT2) and then by DGAT. Then, chylomicrons are secreted into lymphatic vessels and incorporated with triacylglycerol. After catalysis by lipases, the FFAs are stored in adipose tissue or utilized by muscle tissue as an energy source. The remnants of CM are transported into the liver. There, they form triglycerides and are packaged into VLDL particles, which are released into the bloodstream (Fig. 2a) [68, 69].

### Glucose and fructose metabolism in NAFLD

**Fig. 3:**
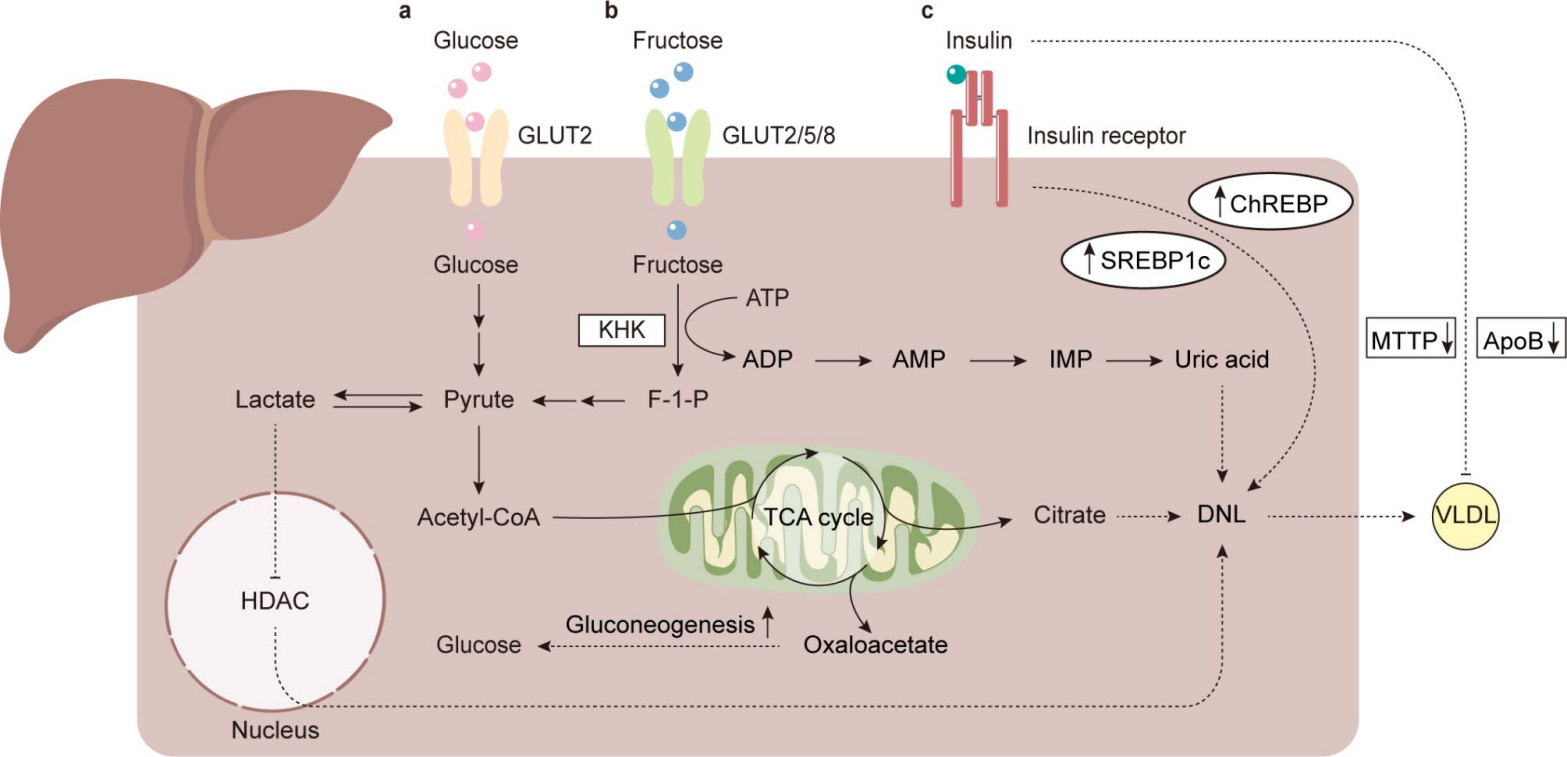
Glucose and fructose metabolism in non-alcoholic fatty liver disease (NAFLD). **a** Increases in glucose transport results in enhanced glycolysis in the liver. There, pyruvate is converted to oxaloacetate, which provides more substrates for de novo lipogenesis (DNL), or lactate, which stimulates the DNL pathway via decreased activity of histone deacetylase (HDAC) [223]. **b** In addition, fructose is phosphorylated to fructose-1-phosphate (F-1-P) by ketohexokinase (KHK) upon entering hepatocytes, which have high-rate activity and bypass more limited steps [224]. Moreover, substrates, such as adenosine diphosphate (ADP) derived from adenosine triphosphate (ATP) during hydrolysis activity, are converted into uric acid, which impairs the liver by stimulating DNL [225, 226]. **c** Insulin regulates the liver directly by upregulating sterol regulatory element-binding protein 1c (SREBP1c) and carbohydrate-responsive element-binding protein (ChREBP); it also decreases the production of very-low-density lipoprotein (VLDL) via the downregulation of microsomal triglyceride transfer protein (MTTP) and apolipoprotein B (ApoB) [78, 79]

Compared with normoglycemic NAFLD patients, hyperglycemic NAFLD patients more rapidly progress from NAFL to NASH [70, 71], indicating that glucose metabolism is tightly associated with NAFLD. Recently, it was found that the levels of key enzymes in glycolysis were significantly higher in NAFLD in parallel with enhanced glycolytic capacity in NAFLD patients. Moreover, overexpression of hexokinase 2 (HK2) and pyruvate kinase isozyme type M2 (PKM2), which are involved in glycolysis, could promote the accumulation of triglycerides in hepatocytes [72, 73]. The Warburg effect produces lactic acids in the presence of oxygen. Tumors often adapt this process, and it also occurs in NAFLD (Fig. 3a) [72]. High levels of lactic acid stimulate the uptake of FFAs by hepatocytes and promote the expression of lipogenic genes [74]. In contrast to the Warburg effect, the effect of the TCA cycle on NAFLD remains controversial. However, there is no doubt that oxidative stress and DNA damage in the NASH stage impair mitochondrial function and worsen the TCA cycle [75].

Insulin resistance is a prominent feature of NAFLD that can regulate NAFLD directly, as evidenced by the observation that the short-term consumption of high-fat diets leads to hepatic insulin resistance without peripheral insulin resistance [76]. Insulin resistance impairs the inhibition of gluconeogenesis [48]. This leads to increased production of glucose [77], which is the main source of DNL. Insulin also promotes DNL by stimulating liver X receptor (LXR), which further upregulates Chrebp1 and Srebp1 [78]. Additionally, insulin inhibits microsomal triglyceride transport protein (MTTP) and promotes apolipoprotein B (ApoB) degradation to regulate VLDL production. In regard to insulin resistance, the increased production of MTTP results from decreased phosphorylation of forkhead box transcription factor 1 (FoxO1) [79] and the degradation of ApoB resulting from the decreased insulin sensitivity and increased uptake of FFAs by the liver (Fig. 3a) [80].

The effect of fructose on NAFLD has also attracted considerable attention recently. Fructose is regarded as the “sweet killer” to metabolic homeostasis [81], and abundant evidence demonstrates that long-term fructose intake aggravates hepatic steatosis [82]. In contrast to glucose, fructose bypasses some regulatory steps in glycolysis. It is catalyzed by phosphofructokinase in the liver and provides more substrates for the DNL pathway [83]. Moreover, the silencing of the feedback cycle in fructose metabolism leads to a continuous decrease in ATP and phosphate [84–87]. This ultimately results in redundant uric acid and deficiency of ATP [88]. Furthermore, ATP deficiency leads to a series of adverse reactions that include inhibitory effects on protein synthesis and oxidative stress [84, 89]. It has also been shown that fructose stimulates the DNL pathway but inhibits β-oxidation by stimulating ChREBP and SREBP1c. This results in a decrease in FFAs consumption [89, 90], thereby worsening NAFLD (Fig. 3b) [84]. In parallel, fructose not only disturbs gut microbiota homeostasis to stimulate hepatic steatosis by regulating the production of short-chain fatty acids (SCFAs) but also destroys tight junctions, which promotes endotoxin exposure to the liver [91–93].

### Gut microbiota in NAFLD

The gut microbiota plays a vital role in barrier protection, immunity and metabolic homeostasis in the host. The main factor that affects the gut microbiota is overnutrition [94]. Gut microbiota dysfunction increases susceptibility to various diseases, including metabolic diseases such as NAFLD [95]. NAFLD is reported to be characterized by chronic low-grade inflammation. Inflammatory mediators, such as endotoxin, are derived from gut microbiota [96], and a high-fat diet increases the proportion of endotoxin [97, 98]. Recent studies on the gut microbiota in NAFLD have found that a high-fat diet increased specific bacteria, such as Enterobacter cloacae B29, Escherichia coli py102 and Klebsiella pneumoniae A7, which impair the progression of NAFLD [99]. Moreover, in regard to the advanced stage, the abundances of Proteus and Escherichia coli were increased, while the abundances of Firmicutes and fecal bacteria were significantly decreased [100]. Additionally, Ruminococcaceae and Veronibacteriaceae were found to be risk factors for liver fibrosis [101]. It has also been found that dysfunction of the gut microbiota dominated by Enterobacteriaceae, Escherichia coli and Shigella is associated with NAFLD progression [102].

**Fig. 4:**
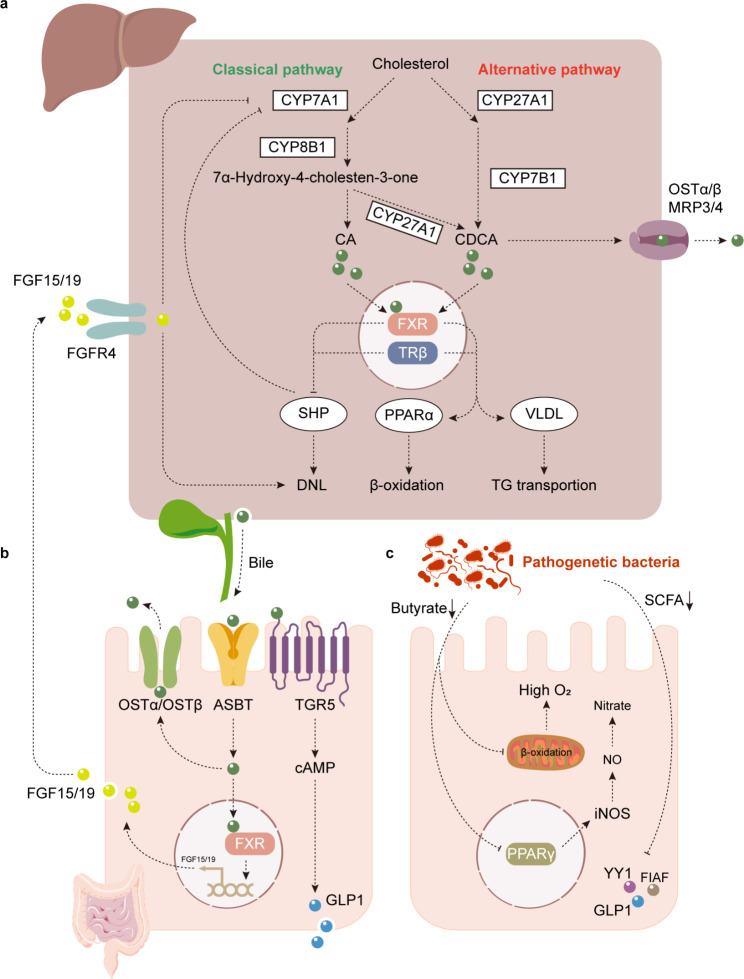
Gut dysbiosis and bile acid metabolism in non-alcoholic fatty liver disease (NAFLD). **a**. Hepatocytes produce primary bile acids via the classic and alternative pathways. The classic pathway starts with cholesterol 7α-hydroxylase (CYP7A1) and the action of sterol 12α-hydroxylase (CYP8B1), which produces cholic acid (CA) or chenodeoxycholic acid (CDCA) through sterol 27 hydroxylase (CYP27A1) [227, 228]. The alternative pathway is initiated by CYP27A1 and produces CDCA through the action of oxysterol 7α-hydroxylase (CYP7B1) [229]. After a meal, the release of cholecystokinin from the pancreas causes bile stored in the gallbladder to be released into the duodenum. Then, ~ 95% of the bile acids involved in the hepatic intestinal circulation are reabsorbed by enterocytes via the apical sodium-dependent bile salt transporter (ASBT) [230] and excreted into the portal vein via organic solute transporter-α and -β (OSTα and OSTβ) [231, 232]. Finally, ~ 5% of bile acids are transported into the systemic circulation from hepatocytes via multidrug resistance-associated protein 3 (MRP3), MRP4, OSTα and OSTβ. **b**. Two kinds of farnesoid X receptor (FXR)-dependent pathways have been proposed for the feedback regulation of bile acid synthesis. Activation of hepatic FXR in the liver increases the expression of the small heterodimer partner (SHP), which inhibits CYP7A1 and CYP8B1 expression [233, 234]. In addition, FXR plays a key role in regulating metabolism in the liver by suppressing de novo lipogenesis (DNL), promoting β-oxidation and producing very-low-density lipoprotein (VLDL) [235–237]. In addition, activation of FXR in the intestine stimulates the production of FGF15/19, which inhibits CYP7A1 and activates the DNL pathway [238]. Another vital receptor for bile acids is Takeda G protein-coupled receptor 5 (TGR5), which promotes the production of glucagon-like peptide-1 (GLP-1) through increased cyclic adenosine monophosphate (cAMP) [239, 240]. **c**. In healthy conditions, the production of butyrate aids in the consumption of oxygen to maintain anaerobic conditions through β-oxidation and decreases the production of nitrate, which is available for specific pathogens via conjunction with peroxisome proliferator activated receptor gamma (PPARγ). Short-chain fatty acids (SCFAs), another beneficial product derived from nondigestible carbohydrates [241], help to maintain metabolic homeostasis through the secretion of GLP-1 and Yin-Yang 1 (YY1) [110, 242]. However, under pathogenic conditions, decreased butyrate and SCFA levels disturb metabolic homeostasis

A number of studies have demonstrated that metabolic dysfunction is associated with decreased concentrations of bacteria that produce SCFAs, propionate and butyrate [103]. On the one hand, butyrate could act as a substrate to stimulate β-oxidation to maintain the anaerobic environment for the microbiota [104] and suppress the expression of nitric oxide synthase via nuclear receptor peroxisome proliferator-activated receptor gamma (PPARγ). This results in a decrease in NO, which inhibits Enterobacteriaceae [105, 106]. On the other hand, butyrate can moderate inflammatory conditions by activating immune cells, such as regulatory T cells (Tregs) [107]. In addition, SCFAs are beneficial for maintaining intestinal permeability and insulin secretion and sensitivity via increased secretion of glucagon-like peptide-1 (GLP-1) and peptide YY (PYY) (Fig. 4c) [108–110]. Unfortunately, dysfunction of the gut microbiota aggravates NAFLD due to a decrease in SCFAs [111]. Specifically, *F. prausnitzii (Faecalibacterium), A. muciniphila (Akkermansia)* and *Dysosmobacter welbionis* are involved in this decrease in SCFAs [112]. Moreover, disorder of the gut microbiota inhibits intestinal epithelial cells from secreting a lipoprotein lipase inhibitor, fasting-induced adipose factor (FIAF), which increases FFAs levels in the liver [28].

### Bile acids metabolism in NAFLD

Systemic homeostasis is influenced by the gut microbiota, partially by regulating bile acids (BAs) metabolism and signal transduction via BAs receptors [113]. Studies have shown that BAs metabolic disorder could aggravate chronic liver diseases [114], and BAs metabolic disorder progresses to NAFLD independent of obesity and diabetes [115]. These findings show the importance of the regulation of BAs in NAFLD. Approximately 95% of BAs are involved in enterohepatic circulation, while the remaining 5% are excreted in the feces [116]. To maintain the BAs pool, the number of newly synthesized BAs should be equal to that of BAs excreted in the feces. Therefore, inhibiting the reabsorption of BAs will increase the excretion of BAs in the feces. Thus, more cholesterol will be converted to BAs, which lowers the risk of obesity [117].

There are two synthesis pathways of BAs. The first is the canonical pathway, also named the neutral pathway (75%), which is regulated by CYP8B1 after cholesterol is hydroxylated by CYP7A. Another pathway is the alternative pathway, also named the acidic pathway (25%). This pathway is controlled by CYP7B1, which is triggered by CYP27A1; as a result, mainly CDCA is produced [118]. It has been reported that activation of the alternative pathway produces more BAs, which benefits the consumption of cholesterol [119]. Additionally, significant increases in CYP8B1 in db/db mice and the overexpression of CYP8B1 have been shown to upregulate lipogenesis-related genes, and this process is dependent on SREBP1. However, the loss of CYP8B1 could ameliorate NAFLD [120, 121].

Moreover, BAs could directly regulate hepatic metabolism as a signal molecule through the activation of farnesoid X receptor (FXR). Hepatic FXR inactivates the lipogenesis pathway by inhibiting SREBP1c. It also induces β-oxidation by activating peroxisome proliferator-activated receptor-α (PPARα) and clears VLDL in plasma, ultimately ameliorating NAFLD [122–124]. Moreover, hepatic FXR stimulates FFAs oxidation and ketogenesis, which is dependent on fibroblast growth factor 21 (FGF21) [125, 126]. However, the activation of intestinal FXR stimulates intestinal epithelial cells to secrete FGF15/19 into the liver, which potently reduces hepatic steatosis and improves insulin resistance [127–130]. However, the contribution of FXR to NAFLD is still under debate due to its wide distribution in various tissues. Recently, it was found that when FXR was globally knocked out, the insulin sensitivity of ob/ob and HFD mice was improved. This may be because the long-term activation of FXR reduces energy consumption and aggravates HFD-induced glucose intolerance (Fig. 4a) [131–133]. However, in liver-specific FXR knockout mice, the above effect was not observed, indicating that intestinal FXR contributes significantly [134]. In parallel, increases in level of T-β MCA, an intestinal FXR antagonist, ameliorates NAFLD through increased BAs synthesis [135–137], and GLP-1 secretion decreases via the activation of intestinal FXR [138]. As a result, the coordination of intestinal FXR in maintaining metabolic homeostasis still needs to be further confirmed (Fig. 4b).

Another bile acid receptor, Takeda G protein-coupled receptor 5 (TGR5), is mainly expressed in the gallbladder, adipose tissue, intestine, and liver and is activated primarily by secondary BAs [139]. Once TGR5 is activated in muscles or brown adipose tissue, it stimulates energy consumption, and in the intestine, it increases the secretion of GLP-1 (Fig. 4b) [114, 140, 141]. Moreover, recent studies found that TGR5 prefers to influence NAFLD-related hypothyroidism regardless of the level of thyroid hormone [142], and researchers found that thyroid hormone β receptor (TRβ) regulates the synthesis of BAs by interfering with SHP [143, 144] or CYP7A1 directly in the liver [145]. Additionally, it has been reported that activation of TRβ reduces systemic lipid content and increases lipid oxidation to improve hepatic lipid homeostasis [146].

## Treatments for NAFLD

### Diet and lifestyle intervention

Several recent studies have demonstrated that steatohepatitis improves in 58% of cases in which the patient lost > 5% of their body weight and in 90% of cases in which the patient lost > 10% of their body weight [10]. Patients are encouraged to adapt a diet pattern of low-fat, low-carbohydrate or Mediterranean type, with a daily energy intake of 500–1000 kcal. It has also been demonstrated that isocaloric diets with high protein content could reduce hepatic steatosis and inflammation in T2DM patients [147].

### Exercise

Exercise has been demonstrated to reduce hepatic steatosis independently of diet changes [148]; additionally, exercise has also been found to improve liver stiffness [149]. Over the course of five years of follow-up, moderate-vigorous exercise was shown to prevent fatty liver in 233,676 subjects who participated in this study [150]. Specifically, a dose‒response relationship was demonstrated between exercise volume and reduction in hepatic steatosis, with individuals exercising over 250 min a week experiencing higher responses [151]. In terms of the type of exercise, sufficient exercise could ameliorate NAFLD regardless of whether aerobic exercise is performed [152].

### Bariatric and metabolic surgery

To date, there is debate regarding the adaptation of foregut bariatric surgery to NAFLD treatment [8], and surgery is only provided for NAFLD patients with other severe obesity-related comorbidities [153]. After surgery, 75% of patients with steatohepatitis showed improvements in ballooning and lobular inflammation [154]. However, the risk of potential complications of secondary steatohepatitis and liver fibrosis is increased [155].

## Updated metabolism-targeted drugs for NAFLD

As the most prevalent chronic liver disease, there is an urgent need for available drugs approved by the FDA for the treatment of NAFLD. In the following, we summarize the emerging pharmacotherapeutic targets and related clinical experimental information regarding metabolic interventions globally (Table 1).

**Table 1:** Emerging metabolic pharmacotherapies for NAFLD globally

Drug target	Drugs in trial	Study in population	Outcomes reported	Reference
ACC inhibitor	Firsocostat	NASH	↓ Hepatic lipid content, ↓ fibrosis	[156]
	PF-05221304	NASH	↓ Steatosis, ↓ ALT/AST, ↑ Hypertriglyceridema	[157]
FASN inhibitor	TVB 2640	NASH	↓Hepatic lipid content, ↓ ALT, ↓ LDL-C, ↓ Fibrosis	[158]
SCD1 inhibitor	Aramchol	NASH	↓ Hepatic lipid content, ↓ ALT, ↓ Fibrosis	[159, 160]
DGAT inhibitor	PF-06865571	NASH	↓ Hepatic lipid content	[163]
MGAT2 inhibitor	BMS-963,272	NASH (Cynomolgus monkeys)	↓ Inflammation, ↓ Fibrosis	[168]
Hypolipidemic Drugs	Atorvastatin	Hypercholesterolemia with hepatic damage	↓ Hepatic lipid content, ↓ Hepatic enzymes	[171]
	Rosuvastatin	NASH	↓ ALT/AST, ↓ Fibrosis	[218]
FGF21 analogue	Pegbelfermin	NASH	↓ Hepatic lipid content, ↑ Insulin sensitivity	[177]
	B1344	NAFLD (Cynomolgus monkeys)	↓ Hepatic lipid content, ↓ Steatosis, ↓Inflammation, ↓ Fibrosis	[179]
PPAR agonist	Pioglitazone	NASH	↓Hepatic lipid content, ↓ ALT/AST	[184]
	Elafibranor	Abdominally obese insulin-resistant males	↓ ALT/AST, ↑ Insulin sensitivity	[188]
	Saroglitazar	NASH	↓ Hepatic lipid content	[219]
	Lanifibranor	NASH	↓ Hepatic lipid content, ↓Inflammation, ↓ Fibrosis	[189]
SGLT-2 inhibitor	Dapagliflozin	Type 2 diabetes	↓ Hepatic lipid content	[192]
	Empagliflozin	NAFLD	↓ Hepatic lipid content, ↓ ALT/AST	[191]
GLP-1 modulator	Liraglutide	NASH	↑ Insulin sensitivity, ↓ NAS score, ↓ ALT/AST	[199]
	Exenatide	NASH	↓Hepatic lipid content, ↓ ALT/AST, ↓ Inflammation	[196]
DPP4 inhibitor	Sitagliptin	NASH	No effect on NAS score	[206]
KHK inhibitor	PF-06835919	NASH	↓ Hepatic lipid content	[209]
Probiotics		NAFLD	No effect on hepatic lipid content and inflammation	[220]
Fecal transplantation		NAFLD	No effect on hepatic lipid content and inflammation	[210]
FXR agonist	Obeticholic acid	NASH	↓ Steatosis, ↓ Inflammation, ↓ Fibrosis	[212]
	Cilofexor	NASH	↓ Hepatic lipid content, ↓ Steatosis, ↓ Primary BAs	[213]
	EDP-305	NASH	↓ Hepatic lipid content, ↓ ALT	[214]
TRβ agonist	Resmetirom	NASH	↓ Hepatic lipid content, ↓ ALT/AST	[215]
FGF19 analogue	Aldafermin	NASH	↓ Hepatic lipid content, ↓ ALT/AST	[217]

### Regulating lipid metabolism

#### ACC inhibitors

Firsocostat, an ACC inhibitor, effectively reduces lipid accumulation and improves fibrosis by inhibiting the DNL pathway after 12 weeks of intervention, but it increased the risk of hypertriglyceridemia [156]. In addition, PF-05221304, developed by Pfizer, is another potent and reversible dual ACC1/2 inhibitor. In a 16-week phase II clinical trial, at least 10 mg of this drug per day dose-dependently reduced lipid accumulation in the liver. The highest percentage of reduction was 65%, but the adverse effect was a dose-dependent increase in triglycerides in serum in 8% of subjects [157].

#### FASN inhibitors

TVB 2640 is an inhibitor of FASN. Patients were randomly divided into groups that received placebo or 25 mg or 50 mg of the drug orally every day for 12 weeks in a phase II clinical trial. Lipid accumulation increased by an average of 4.5% compared to baseline in the control group. However, lipid accumulation was decreased by 9.6% in the TVB 2640-25 mg group and decreased by 28.1% in the 50 mg group. Additionally, the ALT levels decreased in a dose-dependent and time-dependent manner. Moreover, serum LDL levels were decreased in the groups receiving the drug, and no drug-related toxicity was observed in organs. However, this study is limited by the small sample size, and further evaluation of liver histology is needed [158]. Currently, another IIb clinical trial is recruiting volunteers for further evaluation.

#### SCD1 inhibitors

Aramchol, an inhibitor of hepatic stearoyl-CoA desaturase (SCD1), can reduce steatosis, steatohepatitis and liver fibrosis in rodents. Moreover, in a phase II clinical trial, aramchol improved NAFLD, with a 12.5% reduction in hepatic lipid accumulation after 3 months of treatment [159]. Additionally, in a phase IIb clinical trial with more participants, a double-blind trial of 600 mg/per day for 52 weeks, individuals with NAFLD receiving drug intervention showed a 16.7% reduction in hepatic lipid accumulation compared to only a 5% reduction in the placebo group. Moreover, a 29.1% decrease in serum ALT less and a marked improvement in fibrosis less than 1 grade were observed. However, these differences did not reach statistical significance. This drug is considered safe to use because the probability of adverse events is less than 5%. However, the decrease in hepatic lipids was not robust enough, and the differences were not statistically significant [160]. The drug is currently undergoing phase III clinical trials, but outcomes have yet to be reported.

#### DGAT inhibitors

At the end of triglyceride synthesis, DGAT catalyzes the conversion of DAG to triglycerides. This enzyme is classified into two isoforms: DGAT1 and DGAT2. The isoforms have different expression patterns and substrate specificities [161]. Liver-specific DGAT2-deficient mice exhibited reduced hepatic lipid accumulation compared to normal mice [162], and PF-06865571 (a DGAT2 inhibitor) was also shown to reduce the accumulation of lipids in the liver in a phase I clinical trial. Unfortunately, PF-06865571 increases the risk of diarrhea [163]. Currently, another phase II clinical trial has recruited volunteers [164].

#### MGAT2 inhibitors

It has been reported that MGAT2 is overexpressed in the small intestine and liver [165, 166]. Considering the redundancy of the MGAT2 enzyme system, selective inhibition of MGAT2 will only partially impede triacylglycerol synthesis in the intestine. Therefore, this will delay the absorption of fat rather than prevent it completely. As a result, the inhibitor diminishes the risk of diarrhea and other side effects associated with lipid synthesis targets. Moreover, the use of this inhibitor benefits NASH indirectly through weight loss. It has been proposed that MGAT2 contributes to the accumulation of endogenous cannabinoid 2-arachidonoylglycerol, which exhibits anti-inflammatory and antifibrotic effects [167]. Recently, a new selective MGAT2 inhibitor, BMS-963,272, showed benefits in improving liver inflammation and fibrosis without diarrhea in NASH mice. Moreover, BMS-963,272 decreased body weight and increased GLP-1 and PYY levels without adverse effects in a phase I trial [168].

#### Statins

Hyperlipidemia is characterized by increases in triglyceride-rich and cholesterol-rich lipoproteins in the serum. Hyperlipidemia plays a critical role in promoting NAFLD by increasing the transport of lipids to the liver. It has been reported that in prospective clinical trials, statins reduced the risk of hepatic steatosis and fibrosis [169]. Moreover, in a randomized clinical trial, a significant improvement in NAS evaluation after drug treatment was observed in patients with NAFLD [170]. Another small pilot prospective clinical trial demonstrated that the hypolipidemic drug atorvastatin decreases the level of ALT and improves hepatic steatosis [171]. Rosuvastatin also reduces ALT and AST levels and ameliorates liver fibrosis [172]. However, large clinical trials for statins are currently underway to confirm these benefits.

### Hypoglycemic drugs and targeting intermediary metabolism of glucose

#### PPAR agonists

There are three types of PPARs, PPAR-α, PPAR-δ and PPAR-γ, that regulate lipid and glucose metabolism; agonists of PPARs have been shown to ameliorate NAFLD [173]. PPAR-γ greatly regulates adipocyte differentiation and lipid and glucose metabolism and inhibits inflammation [174]. Thiazolidinediones are potent activators of PPAR-γ that are used for the treatment of diabetes, and a further benefit is their ability to reduce plasma FFAs and hepatic lipid accumulation by improving insulin sensitivity [175]. Additionally, thiazolidinediones have been shown to improve fibrosis by directly inhibiting the activation of hepatic stellate cells [176]. Pioglitazone is a mild PPAR-γ activator that ameliorates steatosis and reduces liver enzymes without affecting fibrosis [177]. However, its use is controversial due to the risk of weight gain and edema [178, 179]. This treatment is currently undergoing a phase III clinical trial for treating NAFLD. Elafibranor is a dual agonist of PPARα/δ. It was shown to reduce hepatic lipid accumulation and improve inflammation and fibrosis [180]. When obese patients were treated with elafibranor, liver enzymes decreased and insulin sensitivity improved [181]. However, the latest phase III trial was terminated in advance because the predefined primary surrogate efficacy endpoint was unmet. The dual agonist of PPARα/γ, saroglitazar, significantly reduced hepatic lipid accumulation in mice and is currently used for the treatment of diabetic dyslipidemia in India [182]. However, clinical trials for its use for NAFLD are currently recruiting participants. The pan-PPAR agonist lanifibranor decreased hepatic lipid accumulation, liver enzyme levels, and biomarkers of inflammation in plasma and improved fibrosis in an IIb clinical trial. However, the adverse effects of gastrointestinal reactions and weight gain were greater than those in the control group [182]. A phase III trial is currently recruiting volunteers.

#### Sodium-dependent glucose transporters-2 (SGLT-2) inhibitors

SGLT-2 is a glucose transporter that is dependent on sodium and is responsible for most glucose reabsorption after filtration in the kidney [183]. Because it is not expressed in the liver [183], SGLT-2 indirectly decreases hepatic lipid accumulation through weight loss or metabolic improvement. Additionally, the SGLT-2 inhibitor dapagliflozin reduces hepatic lipid accumulation without significant effects on insulin sensitivity [184, 185]. In patients with type 2 diabetes, empagliflozin reduces liver enzyme levels in plasma and reduces the hepatic accumulation of lipids. It is considered an early treatment for type 2 diabetes patients with NAFLD [186], and it simultaneously reduces the risk of lower extremity amputation and diabetic ketoacidosis [187].

#### GLP-1 modulators

GLP-1 is an endogenous gut hormone that stimulates insulin production and release directly. It also inhibits glucagon secretion indirectly and reduces appetite. GLP-1 receptors are widely distributed but not significantly expressed in the liver [188]. In addition, the improvement in NAFLD by GLP-1 correlates with weight loss and other metabolic improvements, and the benefit of GLP-1 agonists for NAFLD may be an indirect effect that acts by improving systemic metabolism, such as improved insulin sensitivity and appetite suppression. However, exenatide increases hepatocyte uptake of glucose under oral glucose stimulation, suggesting that it directly affects the liver [189]. Until now, it has been debated whether GLP-1 improves NAFLD by regulating the liver directly. T2DM is currently treated with GLP-1R agonists, such as exenatide and liraglutide [190]. Liraglutide not only improves insulin sensitivity [191] but also ameliorates NAFLD with 39% efficacy [192]. Another GLP-1 receptor agonist, exenatide, stimulates β-oxidation and conversely downregulates genes related to lipogenesis, ultimately improving NAFLD [193, 194]. The phases II clinical trial for this drug has ended [195].

#### Dimethyl peptidase 4 (DPP4) inhibitors

DPP4 is widely expressed on a variety of cell surfaces and selectively cleaves a variety of substrates, including GLP-1, to inactivate and thereby regulate diabetes [196]. A decrease in DPP4 activity increases GLP-1 activity. In patients with NAFLD, DPP4 is elevated and positively correlated with hepatocyte apoptosis and fibrosis [197]. Mice with NASH have been shown to benefit from DPP4 inhibitors, as inflammation and fibrosis of the liver was improved [198]. However, in a phase II trial, the DPP inhibitor sitagliptin failed to reduce hepatic lipid accumulation and NAS assessment [199], which means that it is not a reliable strategy for treating NAFLD.

#### Ketohexokinase (KHK) inhibitors

As the rate-limiting enzyme in fructose metabolism, KHK catalyzes the conversion of fructose to fructose 1-phosphate. Excessive fructose is always accompanied by increased hexokinase levels, impaired fatty acid oxidation, enhanced DNL, aggravated hepatic steatosis and impaired insulin signal transduction [200]. When hexokinase is specifically knocked out in the liver, it will moderate the hepatic damage caused by excessive fructose [201]. In an early clinical trial, the hexokinase inhibitor PF-06835919 decreased hepatic lipid accumulation, but no improvement in insulin resistance was observed [202]. To date, a longer-term phase II RCT of PF-06835919 has been carried out in the NAFLD population.

### Drugs targeting the gut-liver crosstalk

#### Microbiota transplantation

Fecal transplantation has emerged as a treatment option for NAFLD, as the gut microbiota differ between NAFLD patients and healthy people. In a phase II RCT, 21 patients with NAFLD received allogeneic or autologous fecal transplantation through endoscopy, but there was no change in hepatic lipid accumulation after six months [203]. Therefore, the feasibility of fecal transplantation needs further investigation. Of note, more studies acknowledge that the appropriate supplementation of butyrate could improve NAFLD. In a randomized controlled trial, a single dose injection of A. soehngenii to the duodenum in Mets patients showed robust GLP-1 production and peripheral glycemic homeostasis [204].

#### FXR agonists

It was shown that OCA, a classic FXR agonist, reduced inflammation, hepatic lipid accumulation, and liver enzyme activity in NAFLD patients. In an ongoing global phase III RCT, liver fibrosis was significantly improved after 18 months of treatment with 25 mg OCA per day, but there was a mild to moderate incidence of adverse effects, such as pruritus [205]. Cilofexor is another FXR agonist. In a completed phase II RCT, 24 weeks of oral administration of 30 mg of cilofexor per day in NASH patients significantly improved steatosis and reduced the content of primary BAs without significant changes in liver fibrosis. In patients taking 100 mg, however, moderate to severe pruritus was experienced [206]. EDP-305 is another FXR agonist. A phase II RCT showed that the ALT level and hepatic lipid accumulation of NAFLD patients were both decreased after 12 weeks of treatment with EDP-305, but the incidence of side effects, including pruritus and nausea, was also higher [207].

#### TRβ agonists

Resmetirom is an oral TRβ agonist that specifically targets the liver to ameliorate NAFLD by improving lipid metabolism and lipotoxicity. In a 36-week phase II RCT, patients receiving 80 mg resmetirom per day had significantly reduced hepatic lipid accumulation, but transient mild diarrhea and nausea were also more common [208]. At present, a phase III RCT for its use as a treatment for NAFLD is recruiting worldwide.

#### FGF19 analogs

Aldafermin is an analog of FGF19 that inhibits BAs synthesis and regulates metabolic homeostasis. In a 24-week phase II RCT conducted in patients with NASH, the results showed that hepatic lipid accumulation decreased by 7.7%, and liver fibrosis trended toward improvement after treatment with aldafermin in NAFLD patients [209]. Another phase IIb RCT revealed that aldafermin was well tolerated, but there was no significant dose-dependent response in fibrosis [210]. Presently, another clinical trial is underway to further support this hypothesis.

#### FGF21 analogs

Fibroblast growth factor 21 (FGF21) is the most prominent hepatokine. It regulates overall metabolic homeostasis by targeting multiple tissues, and its production is highly dependent on nutritional stress, including starvation, a high-fat diet and a nutritional restriction diet [211, 212]. It has been reported that FGF21 exerts beneficial effects in treating obesity due to the potential for increased energy consumption and insulin sensitivity [213], which therefore indirectly benefits hepatic metabolism. Surprisingly, FGF21 has also been reported to directly improve NAFLD, even though the specific mechanism is still unclear [214]. Thus, it is regarded as a promising target for NAFLD. There is a PEGylated analog of FGF21 known as pegbelfermin (PGBF). In a phase II trial, hepatic lipid accumulation in NAFLD patients decreased significantly after subcutaneous injection with PGBF for 16 weeks. While the histology of the liver was still under evaluation, 16% of patients presented adverse effects, such as nausea [215]. Another phase IIb RCT to evaluate the effect of PGBF on fibrosis in NAFLD has ended, but the results have not been reported [216]. Additionally, for 11 weeks, subcutaneous injection of B1344 (another analog of FGF21) significantly reduced hepatic steatosis, inflammation and fibrosis in cynomolgus monkeys suffering from nonalcoholic fatty liver disease (NAFLD), and an evaluation of FGF21 analog administration in nonhuman primate species undergoing liver biopsies for the treatment of NAFLD is first reported in this study [217].

## Conclusion

The threat of NAFLD to human health is gradually increasing. However, to date, there is a lack of specific drugs for treating NAFLD; thus, researchers need to continue to explore potential targets of NAFLD. The results of many studies show that NAFLD patients suffer from diverse metabolic disorders, including lipid, glucose and BAs disorders, which further aggravate NAFLD. The inseparable relationship between metabolism and NAFLD shows the necessity for metabolic therapy. Here, we described the characteristics of lipid metabolism, glucose metabolism, the gut microbiota and BAs metabolism in NAFLD. Various metabolites, including intermediates during the process, can affect the corresponding signaling pathways as signaling molecules. Moreover, different metabolic pathways can act independently or interact with each other to affect NAFLD. The systemic metabolic complexity of NAFLD implies the risk of systemic adverse effects and reveals the challenge of its treatment. Over the past few years, drugs have been tested in clinical trials worldwide. We summarized the therapeutic targets of NAFLD and the corresponding drugs. Due to the complexity of NAFLD, targeted drugs have the defect of a single function. Additionally, a single target has the adverse effect of activating a variety of signaling pathways. As a result, no specific drug is currently available for the treatment of NAFLD. However, from a positive point of view, the metabolic complexity of NAFLD also provides researchers with a combination of drugs and tissue-targeted specific strategies. Currently, clinical trials of multitarget combination therapy and more in-depth investigations in specific tissues of known targets have been ongoing globally. Such studies include GLP-1 receptor agonists combined with DPP4 inhibitors.

It should be noted that NAFLD not only has metabolic dysregulation but also relates to the immunity closely, which could provide aims at the immunotherapy such as the anti-inflammatory and anti-fibrosis agents. Moreover, the beneficial immune factors also could ameliorate NAFLD. Furthermore, the genetic and epigenetic factors have been proved to promote the progression of NAFLD, providing the new therapeutic strategies including RNAi or mRNA vaccines to ameliorate NAFLD. Additionally, we can’t ignore that NAFLD is a whole metabolic homeostatic disease which is link with other diseases, so it is in need for us to detail the underlying mechanisms and find more specific crosstalk factors, which could greatly provide the new targets or therapeutic strategies. Additionally, we should consider using targeted drugs for other closely related diseases in combination with targeted drugs for NAFLD Meanwhile, despite numerous drugs have showed potential in NAFLD in preclinical research, they still fail to achieve the great outcomes in clinical trials, suggesting us revise the experimental models and test strategies to recapitulate the NAFLD pathology in human as realistic as possible, which could tremendously accelerate the drug development of NAFLD. These studies could bring new hope for overcoming NAFLD.

## Electronic supplementary material

Below is the link to the electronic supplementary material.

Supplementary Material 1. List of abbreviations

## Data Availability

Not applicable.
