# Supplementary material for: Pharmacotherapies of NAFLD: updated opportunities based on metabolic intervention
Source: Nutr Metab (Lond). 2023 Jul 6;20:30. doi: 10.1186/s12986-023-00748-x (PMC10327300; doi:10.1186/s12986-023-00748-x)
Supplement: Supplementary file 1 — Supplementary Material 1. List of abbreviations [file 12986_2023_748_MOESM1_ESM.docx]

**List of abbreviations:**

**NAFLD:** Non-alcoholic fatty liver disease；ALT: alanine aminotransferase; AST: aspartate aminotransferase; NAFL: non-alcoholic fatty liver; T2DM: type 2 diabetes mellitus; TGs: Triglycerides; FFAs: free fatty acids; ROS: reactive oxygen species; VLDL: very low-density lipoprotein; cAMP: cyclic adenosine monophosphate; PKA: protein kinase A; p-HSL: phosphorylate lipases phospho-hormone sensitive lipase; p-PLIN1: phospho-perilipin 1; DNL: de novo lipogenesis; SREBP1c: sterol response element binding protein; ChREBP: carbohydrate response element binding protein; FAS: fatty acid synthase; ACC: acetyl-CoA carboxylase; SCD1: stearoyl-CoA desaturase 1; ACP: Acyl carrier protein; LPA: lysophosphatidic acid; GPAT: glycerol-phosphate acyl transferase; PA: phosphatidic acid; PAP: phosphatidic acid phosphorylase; DG: diacylglycerol; DGAT: diacylglycerol acyltransferase; CM: chylomicron; MGAT2: monoacylglycerol acyltransferase 2; HK2: hexokinase 2; PKM2: pyruvate kinase isozyme type M2; LXR: liver X receptor; MTTP): microsomal triglyceride transport protein; ApoB: apolipoprotein B; FoxO1: forkhead box transcription factor 1; SCFAs: short-chain fatty acids; PPARγ: peroxisome proliferator-activated receptor gamma; Tregs: regulatory T cells; GLP-1: glucagon-like peptide-1; PYY: peptide YY; FIAF: fasting-induced adipose factor; BAs: bile acids; FXR: farnesoid X receptor; PPARα: peroxisome proliferator-activated receptor-α; FGF21: fibroblast growth factor 21; TGR5: Takeda G protein-coupled receptor 5; TRβ: thyroid hormone β receptor; SCD1: stearoyl-CoA desaturase 1; SGLT-2: Sodium-dependent glucose transporters-2; DPP4:Dimethyl peptidase 4; KHK: Ketohexokinase; PGBF: pegbelfermin;
